# Risk factors of distant brain failure for patients with newly diagnosed brain metastases treated with stereotactic radiotherapy alone

**DOI:** 10.1186/1748-717X-6-175

**Published:** 2011-12-19

**Authors:** Xiu-jun Chen, Jian-ping Xiao, Xiang-pan Li, Xue-song Jiang, Ye Zhang, Ying-jie Xu, Jian-rong Dai, Ye-xiong Li

**Affiliations:** 1 Department of radiation oncology, Cancer Institute and Hospital, Chinese Academy of Medical Sciences, Peking Union Medical College, Beijing 100021, China

**Keywords:** stereotactic radiotherapy, brain metastasis, distant brain failure

## Abstract

**Objective:**

To explore the risk factors of distant brain failure (DBF) for patients with brain metastasis (BM) who were treated with stereotactic radiotherapy alone and to group the patients on the basis of their risk levels.

**Methods and Materials:**

We retrospectively analyzed 132 newly diagnosed BM patients who were treated with stereotactic radiotherapy alone from May 2000 to April 2010. Kaplan-Meier and Cox proportional hazards regression analyses were performed for univariate and multivariate analyses.

**Results:**

The 1-year incidence rate of DBF was 44.7%, and the median DBF time (MDBFT) was 18 months. In multivariate analysis, the risk factors of DBF were the number of BMs greater than 1 (p = 0.041), uncontrolled extracranial disease (p = 0.005), interval time (IT) of less than 60 months between the diagnosis of primary tumor and BM (p = 0.024), and total volume of BM was greater than 6 cc (p = 0.049). Each risk factor was assigned 1 score. The median survival times for the patients with scores of 0-1, 2-3, and 4 were 31, 12, and 10 months, respectively, and the corresponding MDBFTs were not reached, 13, and 3 months, respectively, (p < 0.001). The crude DBF incidence rates in patients with scores of 0-1, 2-3, and 4 were 14.8%, 50.0%, and 76.9%, respectively, (p < 0.001).

**Conclusions:**

The patients with scores of 0-1 had a lower risk of DBF than the patients with higher scores did, and it may be reasonable to treat these patients with SRS alone and resort to whole-brain radiation therapy only for salvage. The patients with a score of 4 had the highest risk of developing DBF after stereotactic radiotherapy alone, these patients may be candidates for initial whole-brain radiation therapy or clinical trials. The patients with a score of 2-3 had a moderate risk of developing DBF, SRT alone combined with close clinical monitoring would be the optimal treatment regimen for such patients, and for those patients with difficulties in receiving close clinical mornitoring, SRT combined with WBRT will be more suitable.

## Introduction

The initial treatment for newly diagnosed brain metastasis (BM) is controversial. Before the invention of computed tomography (CT), clinicians had no option but to treat the whole brain in cases of BM. The Radiation Therapy Oncology Group (RTOG) [1-4] conducted multiple trials from the 1970s to the 1990s, in which whole-brain radiation therapy (WBRT) for BM was intensively studied; the trials showed that the median survival time (MST) for these patients with BM was only 3-6 months. Since the 1980s, stereotactic radiotherapy (SRT) became widely available, and there are more options for the treatment of BM. Prospective clinical trials on SRT for BM showed that the survival time of BM patients treated with SRT alone was similar to or better than that of patients treated with SRT+WBRT [5-7]. However, whether SRT alone or SRT+WBRT is the optimal regimen for BM patients is yet to be determined. The researchers who are in favor of SRT as the sole initial therapy claimed that WBRT could be avoided in BM patients who initially underwent SRT and that the cognitive function of the patients treated with SRS alone was significantly better than that of the patients treated with SRT+WBRT [5]. On the other hand, the researchers who are in favor of SRT+WBRT as the initial therapy claimed that although the survival times of the patients treated with SRT alone are similar to those of the patients treated with SRT+WBRT, salvage treatment for new intracranial lesions needs to be performed significantly more often in the patients treated with SRT alone than in the patients treated with SRT+WBRT.

BM patients are a heterogeneous group; they show differences in number of BMs, total volume of BM, extracranial disease state, interval between diagnosis of the primary tumor diagnosis of and BM, Karnofsky performance score (KPS), and histologic characteristics. In some studies, these factors were defined as predictors of DBF in patients treated with SRT alone [6,8-12].

Although several studies have included these factors in the secondary analyses, few have attempted to specifically identify the variables that are the predictors of DBF. Therefore, the objective of this study was to identify the risk factors of DBF in patients who were initially treated with SRT alone for newly diagnosed BMs. These variables may help clinicians classify these patients on the basis of their risk of developing new intracranial lesions, which in turn would help clinicians decide whether to resort to WBRT for only salvage or to include it in the initial therapy regimen.

## Methods and Materials

### Patient population

This study included 132 patients with newly diagnosed BM who were treated with SRT alone by using the Linac at the Department of Radiation Oncology, Cancer Institute and Hospital, Chinese Academy of Medical Sciences between May 2000 and April 2010. Cancer and cerebral metastases were confirmed on the basis of histologic analysis of the specimens obtained from extracranial sites, and findings of magnetic resonance imaging (MRI) of the brain, respectively. The demographic and clinical characteristics of the patients are shown in Table 1.

**Table 1:** Patient characteristics

Parameters	Total (n = 132)	No DBF (n = 72)	DBF (n = 60)
**Sex (male %)**	75 (56.8%)	47 (65.3%)	28 (46.7%)
**Age (years)**	57 (27-87)	58 (38-80)	57 (27-87)
**No. of metastases**	1 (1-6)	1 (1-4)	2 (1-6)
1	86 (65.2%)	57 (43.2%)	29 (22.0%)
> 1	46 (34.8%)	15 (11.4%)	31 (23.5%)
**Total target volume (cc)**	4.26 (0.10-57.84)	3.42 (0.23-57.84)	3.45 (0.10-45.47)
> 6	50 (37.9%)	22 (16.7%)	28 (21.2%)
≤ 6	82 (62.1%)	50 (37.9%)	32 (24.2%)
**Extracranial disease**			
Progressive	91 (68.9%)	43 (32.6%)	48 (36.4%)
Absent or controlled	41 (31.1%)	29 (22.0%)	12 (9.1%)
**IT (months)**	12 (0-240)	12 (0-240)	13 (0-192)
≥ 60	20 (15.2%)	15 (11.4%)	5 (3.8%)
< 60	112 (84.8%)	57 (43.2%)	55 (41.7%)
**KPS score**	80 (40-90)	80 (40-90)	70 (50-90)
KPS = 70	45 (34.1%)	18 (13.6%)	27 (20.5%)
KPS ≠ 70	87 (65.9%)	54 (40.9%)	33 (25.0%)
**Histologic characteristics**			
NSCLC	79 (59.8%)	45 (34.1%)	34 (25.8%)
Melanoma	5 (3.8%)	2 (1.5%)	3 (2.3%)
Breast cancer	15 (11.4%)	8 (6.1%)	7 (5.3%)
RCC	9 (6.8%)	6 (4.5%)	3 (2.3%)
Other	24 (18.2%)	11 (8.3%)	13 (9.8%)

IT, interval time; KPS, Karnofsky performance score; NSCLC, non-small cell lung cancer; RCC, renal cell carcinoma

### Study variables

We reviewed the patients' records and obtained data on potential risk factor variables including histologic characteristics, total number of metastases, total volume of metastases, interval time (IT) between the diagnosis of primary tumor and BM, KPS score, and the extracranial disease state. Tumor volume was recorded on the basis of the contouring required in SRT planning. Extracranial disease was defined as absent, present but controlled, or progressive.

### SRT

The XSTPS V2.2 plan system (Creat company, China, May 1, 2000 to July 31, 2008) and BrainSCAN 5.31 plan system (August 1, 2008 to April 30, 2010) were used for SRT in the patients. All the patients were treated as outpatients. The treatment plan was formulated by a radiation oncologist and a radiation physicist. Delineation of gross tumor volume (GTV) was identified using CT and MRI fusion images. To determine the planning target volume (PTV), we used a margin of 2 mm in all directions around the GTV, and 80-90% isodose enclosed the PTV. Dose fractionation schemes were 20-30 Gy/1 f/1 d and 24-50 Gy/5-12 Gy/2-10 f. Fractionated radiation therapy was performed daily or on alternate days, the median total dose was 30 Gy. The radiation dose was contingent on tumor volume and location in the brain, and the SRT fractionation scheme was listed in table 2.

**Table 2:** SRT fractionation scheme

Volume of single lesion	Dose fractionation schemes	Number of lesions
≤ 3 cc	20~30 Gy/1f/1d 45 Gy/5 Gy/9f/11d(2 lesions of brain stem)	78(35.3%)

3 cc~9 cc	24 Gy/12 Gy/2f/2d 30~36 Gy/10~12 Gy/3f/3d 40 Gy/10 Gy/4f/4-7d 40~45 Gy/8~9 Gy/5f/5-7d	120(54.3%)
≥ 9 cc	42 Gy/6 Gy/7f/9d 45 Gy/5 Gy/9f/11d 50 Gy/5 Gy/10f/12d	23(10.4%)

		Total: 221(100%)

### Follow-up

The first follow-up occurred at 1 month after stereotactic radiation therapy, and follow-up was conducted at 2-3 months intervals thereafter. At each visit, a recent brain MRI study was reviewed and compared with previous MRI studies.

### Statistical analysis

The overall survival time was calculated as the time between SRT and death from any cause. For surviving patients, they were censored at the date of the last follow-up. The interval between the end of SRT and the first occurrence of new intracranial lesions, which was determined by using MRI was defined as DBF time. If there was no record of DBF, patients were censored at the date of their last MRI. Kaplan-Meier and Cox proportional hazards regression analyses were used for univariate and multivariate analyses, respectively, for all possible risk factors associated with DBF. The MST and median DBF time (MDBFT) were calculated by using the Kaplan-Meier analysis.

## Result

During our study period, 109 patients died. Of the 23 surviving patients, 9 (39.1%) showed DBF. Fifty (45.9%) of the 109 patients who died showed DBF. This difference was not statistically significant (p = 0.521). The reasons of death were listed in table 3. There were 24(22.0%) patients died of uncontrolled brain metastasis for the whole group, And for the patients who suffered from DBF, 19(35.8%) were died of uncontrolled brain metastasis, but for the patients without DBF, only 6(9.8%) died of uncontrolled brain metastasis. 44.7%. The 6-month and 1-year local control rate was 86.9% and 81.4%, separately.

**Table 3:** Reasons of death

Reason of death	The whole group n = 109	DBF n = 50	NO DBF n = 59
Uncontrolled brain metastasis	24(22.0%)	18(36.0%)	6(10.2%)
Progression of the extracranial disease	71(65.1%)	25(50.0%)	46(77.9%)
Non-cancer related reasons	9(8.3%)	4(8.0%)	5(8.5%)
Unknown reasons	5(4.6%)	3(6.0%)	2(3.4%)

The overall 1-year incidence rate of DBF was 44.7%. Univariate analysis showed that the number of BM (p = 0.003), total target volume (p = 0.021), extracranial disease state (p = 0.003), interval between the diagnosis of primary tumor and BM (p = 0.011), and KPS (p = 0.012) significantly affected the DBF incidence rate; the differences in the histologic characteristics of the 2 groups were not statistically significant in the univariate analysis (Table 4). All potential risk factors were analyzed in the multivariate analysis. Number of BMs (1 vs. 2 or more, p = 0.041), total target volume (≤ 6 cc vs. > 6 cc, p = 0.049), extracranial disease state (absent or stable vs. uncontrolled, p = 0.005), IT between the diagnosis of the primary tumor and BM (≥ 60 months vs. < 60 months, p = 0.024) were independent risk factors for DBF; the differences in KPS and histologic characteristics of the 2 groups were not statistically significant (Table 5).

**Table 4:** Results of the univariate analysis

Parameter	Number of patients	MDBFT (months)	95% confidence interval	p value
No. of metastases				0.003
1	86 (65.2%)	54	--	
> 1	46 (34.8%)	8	2.0-14.1	
Total target volume (cc)				0.021
> 6	50 (37.9%)	10	4.7-15.3	
≤ 6	82 (62.1%)	21	11.8-30.2	
Extracranial disease				0.003
progressive	91 (68.9%)	11	6.3-15.7	
absent or controlled	41 (31.1%)	Not reach	--	
IT(months)				0.011
≥ 60	20 (15.2%)	Not reach	--	
< 60	112 (84.8%)	12	7.6-16.5	
KPS score				0.012
KPS = 70	45 (34.1%)	10	3.3-16.7	
KPS ≠ 70	87 (65.9%)	21	9.5-32.5	
Histologic characteristics				0.762
Melanoma	5 (3.8%)	18	0-41.4	
Others	127 (96.2)	15	7.7-23.3	
Risk factor scores				< 0.001
0-1	27 (20.5%)	Not reach	--	
2-3	92 (69.7%)	13	8.4-17.6	
4	13 (9.8%)	3	2.0-4.0	

IT, interval time; KPS, Karnofsky performance score

**Table 5:** Results of the multivariate analysis

Parameter	p value	Hazard ratio	95% confidence interval for Hazard ratio
**No. of metastases** > 1 vs. 1	0.041	1.77	1.02-3.05
**Total target volume (cc)** ≤ 6 vs. > 6	0.049	0.59	0.35-0.99
**Extracranial disease** progressive vs. absent or controlled	0.005	2.55	1.33-4.87
**IT (months)** ≥ 60 vs. < 60	0.024	0.34	0.14-0.87
**KPS score** ≠70 vs. = 70	0.140	0.66	0.39-1.14
**Histologic characteristics** Melanoma vs. Others	0.416	1.66	0.49-5.58

IT, interval time; KPS, Karnofsky performance score

**Figure 1:**
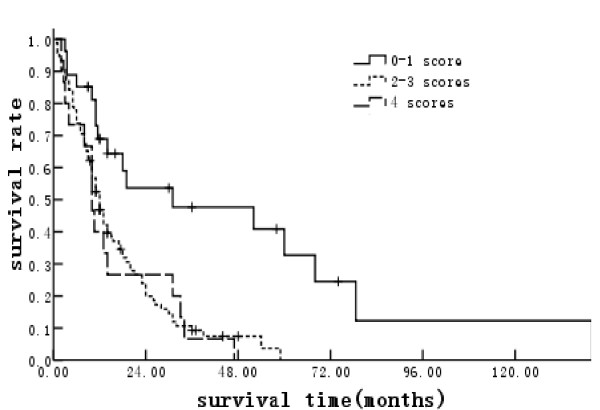
**Median survival time classified according to risk factor scores**.

**Figure 2:**
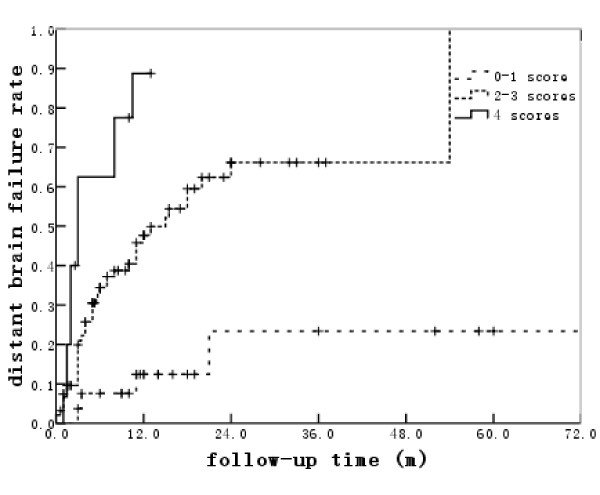
**Median distant brain failure time classified according to risk factor scores**.

Each risk factor with a significance value of p ≤ 0.05 in the multivariate proportional hazard model was assigned a score of 1. The MSTs for patients with scores of 0-1, 2-3, and 4 were 31, 12, and 10 months, respectively (Figure 1). The corresponding MDBFTs after the initial SRT were not reached, 13 and 3 months, respectively (p < 0.001) (Figure 2). The median follow-up time for the whole group was 12 months, and that for the patients with scores of 0-1, 2-3, and 4 were 17.5, 12, and 10 months, respectively. During the follow-up, the crude DBF incidence rates for patients with scores of 0-1, 2-3, and 4 were 14.8%, 50.0%, and 76.9%, respectively (p < 0.001).

## Discussion

Published literature showed that about 20-40% of the BM patients died of intracranial diseases [1,7,13,14], and 60-80% died because of progression of the extracranial disease or other reasons. With the development of cancer therapy, both intracranial and extracranial diseases were controlled better than they were earlier: the overall survival time of BM patients was prolonged, and more importantly, the quality of life of these patients improved.

Thus far, almost all the clinical trials have shown that the MST of BM patients treated with SRT alone is similar to that of the patients treated with SRT+WBRT. The point at issue is the influence of WBRT on the quality of life or neurocognitive functions of these patients.

In 2007, Aoyama [15] et al. reported how the omission of WBRT affected the neurocognitive function of patients with 1-4 BMs who were previously treated with SRT. Neurocognitive function was assessed by using the Mini-Mental State Examination (MMSE), which was criticized for its low sensitivity [16]. The DBF incidence rate in the patients treated with SRT+WBRT group was much lower than that in the patients treated with SRT alone. The average intervals before deterioration of neurocognitive function in patients treated with WBRT+SRT and SRT alone were 16.5 and 7.6 months, respectively, (p = 0.05). However, the neurocognitive function preservation rate, determined on the basis of the MMSE score, was not significantly different between the 2 patient groups. The patients treated with WBRT+SRT showed a more continuous decrease in their MMSE scores than the patients initially treated with SRT. The long-term adverse effects of WBRT on neurocognitive functions may not be negligible. In 2009, Chang et al. [5] reported a randomized clinical trial in which they tested whether the advantages of performing WBRT after SRT for the control of brain tumors outweighs the potential neurocognitive risks of WBRT. Chang et al. evaluated the neurocognitive function by using the Hopkins Verbal Learning Test-Revised (HVLT-R), which is more precise than the MMSE. The recurrence rate of intracranial lesion was higher in the patients treated with SRT alone than in the patients treated with WBRT+SRT. However, the risk of a significant decline in learning and memory function after 4 months was lower in the patients treated with SRT alone than that in the patients treated with WBRT+SRT after effective salvage treatment. As a result, Chang et al. recommended the use of a combination of SRT and close clinical monitoring for the initial treatment of newly diagnosed BM. However, in our trial, salvage treatment for the 2 groups was not the same; the treatment was more aggressive for the patients treated with SRT alone; therefore, some authors questioned the reliability of these results. The European Organization for Research and Treatment of Cancer (EORTC) 22952-26001 [7] study was a randomized phase III trial on patients with 1-3 BMs. Patients were randomly selected for treatment with SRT alone or SRT+WBRT; the primary end point was the functional survival time. Although this trial did not evaluate the neurocognitive functions of the 2 groups, the functional survival time of the 2 groups was similar.

From the above literatures, we can found that WBRT can improve the intracranial tumor control rate and decrease the risk of DBF, but it cannot increase the overall survival time of BM patients; and we can not find any evidence to indicate that a better intracranial tumor control rate can lead to a better quality of life for these patients. Therefore, it is important to determine which patients are at a high risk of DBF after SRT alone; WBRT would be necessary for these patients.

According to the published articles, the possible risk factors of DBF include number of BMs [6,8,10,12], total target volume [11], extracranial disease state [6,8,9,11], interval between the diagnosis of the primary tumor and BM [10], KPS [6], and histologic characteristics [8]. The results of our multivariate analysis suggest that the number of BM, total target volume, extracranial disease state, and the interval between the diagnosis of the primary tumor and BM were independent risk factors of DBF; this finding is similar to those of the above-mentioned articles. The intergroup differences in KPS in the univariate analysis were statistically significant, but those in the multivariate analysis were not. Therefore, KPS was not considered an independent risk factor for DBF. The differences in histologic characteristics of the 2 groups in both univariate and multivariate analyses were not significantly different; this finding could be attributable to the small number of patients with small cell lung cancer (SCLC) and melanoma.

Thus far, several studies have attempted to identify predictors of DBF (or metachronous BM) in secondary analyses, but only Sawrie et al. [8] specifically identified these variables in the primary analyses. In that study BM patients were divided into 2 subgroups: the low-risk subgroup (without risk factor) and the high-risk subgroup (with risk factors) (there was no moderate risk subgroup, which was different from our results). In our study, BM patients were divided into 3 subgroups on the basis of the number of the risk factors: patients with a score of 0-1, patients with a score of 2-3, and patients with a score of 4. The intergroup differences in both MDBFT (not reached, 15, and 3 months; p < 0.001) and crude incidence rates of DBF (14.8%, 50.0%, and 76.9%; p < 0.001) were statistically significant.

Our data suggested that patients with a score of 0-1 had a low risk of developing DBF and that it may be reasonable to treat these patients with SRT alone. These patients would still have the option of undergoing additional SRT or WBRT as salvage therapy, depending on the nature and severity of any disease progression. The patients with a score of 4 were at the highest risk to of developing DBF, such as these patients 4 scores subgroup, may be better candidates for WBRT as a part of their initial treatment or for enrollment into clinical trials. Fifty percent of the patients with a score of 2-3 scores subgroup would develop DBF during their life span. Therefore, we recommend that SRT alone with close clinical monitoring be performed as the initial treatment in these patients in consideration of the palliative intent of BM therapy. However, if in cases in which close clinical monitoring cannot be performed, then combined therapy of SRT+WBRT for BM should be performed as initial treatment. Our treatment recommendations were summarized in table 6.

**Table 6:** Treatment recommendations by risk level

Risk factor score summation	Crude DBF rate	Treatment recommendations
0~1	14.8%,	SRT as initial treatment and WBRT ± SRT as salvage treatment for DBF
2~3	50.0%,	SRT + close clinical monitoring as initial treatment; if close clinical monitoring is impossible, then WBRT+SRT as initial treatment
4	76.9%	WBRT+SRT as initial treatmentt

Risk factors include number of BMs > 1, total target volume > 6 cc, uncontrolled extracranial disease and interval time between the diagnosis of the primary tumor and BM < 60 months, and each risk factor was assigned a score of 1

However, the results of this study are subject to any of the biases inherent in a retrospective analysis, and our risk stratification requires prospective validation. Moreover, since most of the patients included in our study had 1-3 BMs and only 6.8% of the patients had more than 3 lesions, it is unclear whether our findings are applicable for such patients with more than 3 brain metastasis.

## References

[B1] Komarnicky LT Phillips TL Martz K A randomized phase III protocol for the evaluation of misonidazole combined with radiation in the treatment of patients with brain metastases (RTOG 79-16) Int J Radiat Oncol Biol Phys 1991 20 53 58 10.1016/0360-3016(91)90137-S 1993631

[B2] Sause WT Scott C Kirsch R Phase I/II trial of accelerated fractionation in brain metastases, RTOG 85-28 Int J Radiat Oncol Biol Phys 1993 26 653 657 10.1016/0360-3016(93)90284-3 8330997

[B3] Phillips TL Scott CB Leibel S Results of a randomized comparison of radiotherapy and bromodeoxyuridine to radiotherapy alone for brain metastases: Report of RTOG trial 89-05 Int J Radiat Oncol Biol Phys 1995 33 339 348 10.1016/0360-3016(95)00168-X 7673021

[B4] Murray KJ Scott C Greenberg HM A randomized phase III study of accelerated hyperfractionation versus standard in patients with unresected brain metastasis: A report of RTOG9104 Int J Radiat Oncol Biol Phys 1997 39 571 574 10.1016/S0360-3016(97)00341-6 9336134

[B5] Chang EL Wefel JS Hess KR Neurocognition in patients with brain metastases treated with radiosurgery or radiosurgery plus whole-brain irradiation: a randomised controlled trial Lancet Oncol 2009 10 1037 44 10.1016/S1470-2045(09)70263-3 19801201

[B6] Aoyama H Shirato H Tago M Stereotactic radiosurgery plus whole-brain radiation therapy vs stereotactic radiosurgery alone for treatment of brain metastases: A randomized controlled trial JAMA 2006 295 2483 2491 10.1001/jama.295.21.2483 16757720

[B7] Kocher M Soffietti R Abacioglu U Adjuvant Whole-Brain Radiotherapy Versus Observation After Radiosurgery or Surgical Resection of One to Three Cerebral Metastases: Results of the EORTC 22952-26001 Study J Clin Oncol 2011 29 2 134 141 10.1200/JCO.2010.30.1655 21041710 PMC3058272

[B8] Sawrie SM Guthrie Bl Spencer SA Predictors Of Distant Brain Recurrence For Patients With Newly Diagnosed Brain Metastases Treated With Stereotactic Radiosurgery Alone Int J Radiat Oncol Biol Phys 2008 70 1 181 186 10.1016/j.ijrobp.2007.05.084 17768015

[B9] Radbill AE Fiveash JF Falkenberg ET Initial Treatment of Melanoma Brain Metastases Using Gamma Knife Radiosurgery Cancer 2004 101 4 825 833 10.1002/cncr.20447 15305416

[B10] Lutterbach J Cyron D Henne K Ostertag CB Radiosurgery followed by planned observation in patients with one to three brain metastases Neurosurgery 2008 62 2 776 84 18596428 10.1227/01.neu.0000316281.07124.ea

[B11] Yu CH Chen JCT Apuzzo MLJ Metastatic Melanoma To The Brain: Prognostic Factors After Gamma Knife Radiosurgery Int J Radiat Oncol Biol Phys 2002 52 5 1277 1287 10.1016/S0360-3016(01)02772-9 11955740

[B12] Matsunaga S Shuto T Kawahara N Suenaga J Inomori S Fujino HJ Gamma Knife surgery for metastatic brain tumors from primary breast cancer: treatment indication based on number of tumors and breast cancer phenotype Neurosurg 2010 113 1 65 72 10.3171/2010.8.GKS10932 21121788

[B13] Knisely JPS Berkey B Chakravarti A A Phase III Study Of Conventional Radiation Therapy Plus Thalidomide Versus Conventional Radiation Therapy For Multiple Brain Metastases (RTOG 0118) Int J Radiat Oncol Biol Phys 2008 71 1 79 86 10.1016/j.ijrobp.2007.09.016 18164847

[B14] Higuchi Y Serizawa T Nagano O Three-Staged Stereotactic Radiotherapy Without Whole Brain Irradiation For Large Metastatic Brain Tumors Int J Radiat Oncol Biol Phys 2009 74 5 1543 1548 10.1016/j.ijrobp.2008.10.035 19135317

[B15] Aoyama H Tago M Kato N Neurocognitive Function Of Patients With Brain Metastasis Who Received Either Whole Brain Radiotherapy Plus Stereotactic Radiosurgery Or Radiosurgeryalone Int J Radiat Oncol Biol Phys 2007 68 5 1388 1395 10.1016/j.ijrobp.2007.03.048 17674975

[B16] Meyers CA Wefel JS The use of the Mini-Mental State Examination to assess cognitive functioning in cancer trials: No ifs, ands, or buts, or sensitivity J Clin Oncol 2003 21 3557 3558 10.1200/JCO.2003.07.080 12913103

